# Supplementary material for: The Expression of Epac2 and GluA3 in an Alzheimer’s Disease Experimental Model and Postmortem Patient Samples
Source: Biomedicines. 2023 Jul 25;11(8):2096. doi: 10.3390/biomedicines11082096 (PMC10452319; doi:10.3390/biomedicines11082096)
Supplement: Supplementary file 1 [file biomedicines-11-02096-s001.zip › biomedicines-2523330-supplementary.pdf]

biomedicines

Supplementary Materials

Table S1. Characteristics of healthy controls and AD patients.

	**Controls (n=6)**	**AD (n=7)**
Gender, female (n%)	1 (16.67)	2 (28.57)
Age (years), mean (SEM)	80.17 (1.45)	76.21 (3.58)
Braak stage, mean (SEM)	N.D.	4.60 (0.60)
Anti-depressant medication, n (%)	N.D.	1 (16.67)

n, number of patients; SEM, standard error mean; N.D., not determined;
